# A Survey of Caregivers’ Knowledge on Detection and Management of Pediatric Fever

**DOI:** 10.7759/cureus.14222

**Published:** 2021-03-31

**Authors:** Anthony Concilla, Ryan Kovacik, John Kobilis, Megan Stobart-Gallagher

**Affiliations:** 1 Emergency Medicine, Einstein Medical Center Philadelphia, Philadelphia, USA; 2 Emergency Medicine, Baptist Health South Florida, Miami, USA; 3 Emergency Medicine, Thomas Jefferson University Hospital, Philadelphia, USA

**Keywords:** pediatrics, fever, dehydration, caregivers

## Abstract

Introduction

Fever is a common presenting symptom for children accounting for over 20% of visits to the emergency department (ED). The symptom of fever in children has been shown to create undue anxiety amongst caregivers. The purpose of this study was to evaluate caregivers’ detection and management of pediatric fevers to identify potential knowledge gaps in our patient population.

Methods

Caregivers of children aged 3 months to 12 years presenting to an urban, Level I trauma center with various complaints (not limited to fever) were surveyed using an 11-question paper questionnaire. All data was collected anonymously, then correlated and analyzed using Microsoft Excel (Microsoft Corporation, Redmond, WA, USA). The questionnaire elicited information regarding home detection, management, timeframe and location to seek care, and caregiver concerns surrounding fever.

Results

A total of 276 caregiver responses were collected. Overall, 90.9% of subjects had a thermometer at home but the method of taking a temperature ranged. In regards to the caregivers’ definition of fever, 44.4% defined a fever to be at or above 38℃ when measured. When seeking care for a fever, 41% waited less than 24 hours with only 12% waiting more than 48 hours. Many caregivers utilized their pediatrician (45.3%) for fever evaluation, but a large group utilized the ED (26.8%). Dehydration was their most common concern, with seizures, worsening infection, brain damage, and death as the additional reported fears.

Conclusion

Our study found caregiver knowledge gaps in the identification of fever as well as specific concerns that fever would lead to dehydration and severe infection. These concerns lead to seeking care very early in a child's illness in both the emergency department and pediatrician's office. This presents an opportunity for further caregiver education to decrease or alter the timing or location of care sought in a pediatric febrile illness.

## Introduction

Fever can be a normal and even beneficial physiological response to illness in children, yet a statistical analysis by McDermott et al. in 2015 [1] determined there were 30 million emergency department (ED) visits nationally with a chief complaint of fever for children aged 18 and younger, with an overwhelming majority (96.7%) being discharged without admission. Despite the relative innocuousness, fever is an incredibly common reason for visits to pediatricians, with estimates that over 30% of visits there include a complaint of fever [2].

The anxieties surrounding pediatric fever by caregivers were initially studied in 1981 when Dr. Barton Schmitt coined the term “fever phobia” [3]. Numerous investigators have since performed follow-up studies that confirm the persistence of fever phobia in their respective patient populations [4-5]. A global meta-analysis performed in 2019 examined relevant papers published from 1985-2018 and confirmed many pediatric caregivers and even healthcare workers misinterpret fevers leading to overtreatment and unnecessary stress. These findings were evident in 65 papers spanning more than 26,000 caregivers [6]. Socioeconomic status is among the most significant determining factors of caregivers demonstrating fever phobia, as well as lower caregiver education levels and younger age of children [6-7].

The concept of “fever phobia” has continued to exist among caregivers and contributes to the potential for misuse of healthcare resources due to lack of education regarding the benign nature of most febrile illnesses. Caregivers’ misconceptions about the dangers of fever have the potential to lead to mismanagement of the symptom and misuse of healthcare resources. The purpose of this study was to evaluate caregivers’ detection and management of pediatric fevers to identify potential knowledge gaps in our patient population by evaluating their fears, detection methods, and management decisions when a child is faced with a febrile illness. The preliminary data from this work was previously presented as an abstract at a regional Society of Academic Emergency Medicine meeting.

## Materials and methods

This study was a cross-sectional observational study set in a Level 1 trauma center in Philadelphia, PA, USA that sees over 100,000 patient visits annually with approximately 18% of those being patients under the age of 18. It serves a predominantly African American patient population in an area of the city where greater than 30% of the population live below the poverty line set forth in the last data published by the United States Census Bureau in 2018 [8]. The median income at that time was approximately $28,000/annually. The ED has a dedicated research division of full-time employees with medical education backgrounds and specific research training who are available for recruitment 16 hours a day, 7 days a week. We recruited 276 caregivers of patients aged 3 months to 12 years over a 6-month period.

A literature review was completed prior to the development of the survey to determine work previously done in this realm. The Institutional Review Board at our institution determined this study exempt from approval. The study was conducted and data collected from January through June of 2016 and enrollment took place during the hours of 8 am to 11 pm eastern standard time. Inclusion criteria included any primary caregivers present in the ED at the time of a pediatric visit - chief complaints varied and did not have to be specifically fever-related to be approached. All presenting complaints were considered to broaden the population studied beyond those with fever at the time of presentation in the hopes of obtaining generalizable data. A caregiver was defined as the primary guardian and was not limited to biological parents. Exclusion criteria consisted of persons who not the primary caregivers of patients aged 3 months to 12 years, level 1 trauma patients and their visitors, visitors of recently deceased patients (who died in the ED at time of stay), patients under psychiatric hold, patients/guardians who had diminished decision-making capacity, were in police custody, and prisoners. Also, guardians of patients who were otherwise not healthy or with chronic cardiac, neurologic, or immunocompromising conditions and guardians or patients that were admitted/transferred to an admitting facility were excluded.

Prospective subjects were identified in the ED using the electronic medical record by dedicated ED research associates. All of those identifying and recruiting subjects were educated continually on the study protocol and inclusion/exclusion criteria. Research associates did not approach caregivers until it was determined by the treating physicians that the child would not require admission for further management of their presenting complaint. Patients and their caregivers received all standard evaluation and treatment deemed necessary by the treating ED physicians prior to enrollment. 

Caregivers were given an anonymous 11-question paper survey created by our research team to fill out after obtaining verbal consent by research associates. Each caregiver completed the questionnaire one time. Once completed, surveys were stored securely in our research office until data analysis.

**Figure 1:**
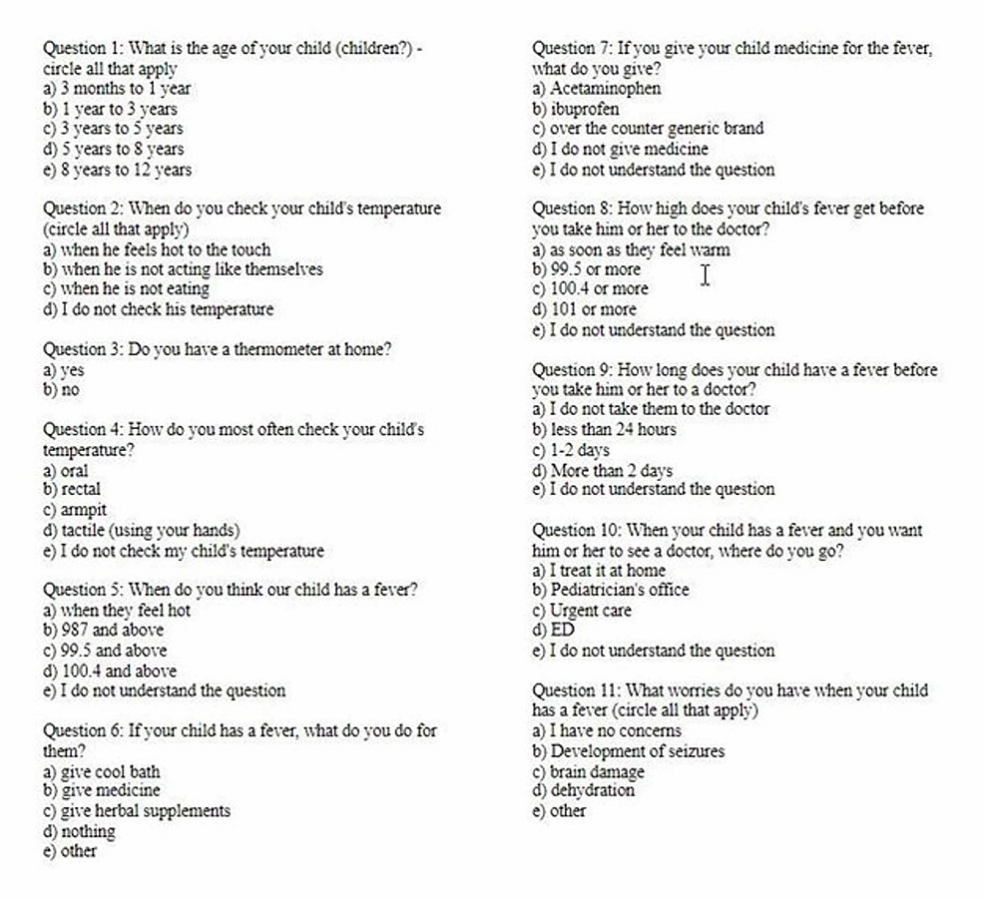
Representative survey given to caregivers

This study was designed to elicit information regarding pediatric caregivers’ breadth of knowledge regarding fever and how they manage the child’s symptoms, when and where they seek care, and eliciting their concerns about fever in their child. The questionnaire specifically addressed information regarding home fever management including use of a thermometer, mode of fever detection, treatment modalities, medication choice, time frame to seek physician care, physician location preference, and largest fears surrounding fever. Surveys responses were collected anonymously and did not request any demographic information. Figure 1 shows the multiple-choice questions included in the survey provided to caregivers. Answer choices were inserted into an excel document and the number of times each answer was marked was recorded. The frequencies of each answer choice were calculated. 

## Results

A total of 276 caregiver responses were collected over 6 months’ time. There was no demographic information collected for the caregivers as the surveys were anonymous. Overall, 90.9% of subjects had a thermometer at home. When checking their child’s temperature across all ages of children included in the study, 35.8% of caregivers most often used the oral method. Of note, 21.9% of caregivers with a child aged six months to one year most often used the rectal temperature. Utilization of both the oral and the rectal method was preferred by 8.0% of caregivers. Armpit was the third most common location for temperature checking with 40 (14.5%) of caregivers preferring this method.

**Figure 2:**
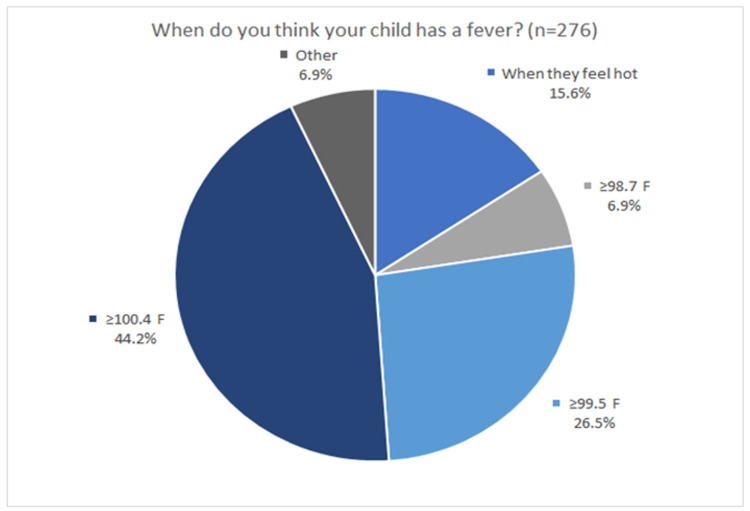
Caregivers' definitions of fever

When asked what prompts a caregiver to initially check a child’s temperature, the majority (33.0%) check the temperature when the child subjectively feels hot. The child’s demeanor was also a determining factor for checking the temperature with 46 (16.7%) caregivers taking a temperature when the child was deemed to not be acting like themselves. Additionally, 61 (22.1%) check when the child simultaneously feels hot and is not acting like themselves, and 51 (18.5%) check when the child has a triad of feeling warm, abnormal behavior, and decreased oral intake. Figure 2 shows how caregivers defined fever in their child. Of the options provided regarding when the caregiver believed their child had a fever, 44.2% (122) believed 100.4°F and above qualified as abnormal. Less than 7.0% (19) believed 98.7°F and above was considered a fever, and 26.5% (73) felt at 99.5°F and above was considered febrile. Interestingly, 15.6% (43) believe their child has a fever “when their child feels hot.”

**Figure 3:**
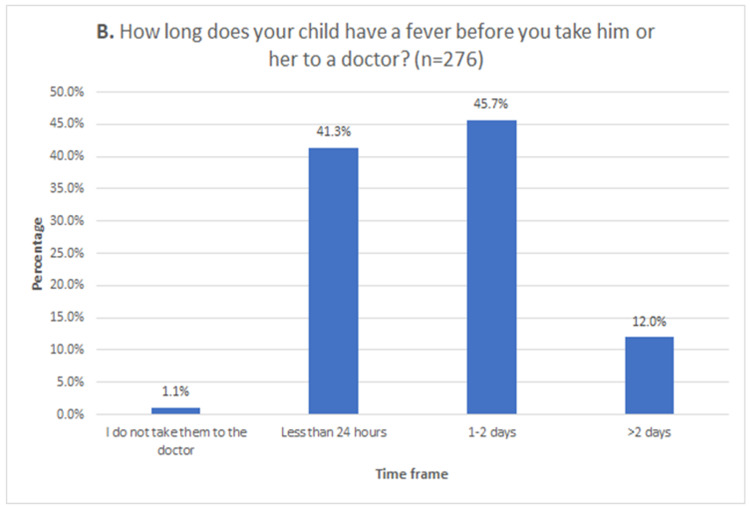
Duration of time caregivers utilize to determine when their child needs to be seen by a physician

Caregivers were asked how high the child’s fever would get before taking him or her to the doctor, and how long they would wait before going to the doctor. The majority (45.3%) responded that they would take their child at a temperature of 100.94°F or above. This was followed by 86 caregivers (31.2%) taking the child with a temperature of 100.4°F or above, and 47 (17.0%) when the child has a temperature of 99.5°F or more. Only 17 (6.2%) will take the child as soon as they subjectively feel warm. The overwhelming majority (87.0%) reported they will take the child to the doctor between 0 and two days of fever onset. Of this majority, 114 caregivers (41.3%) stated they would take their child to the doctor less than 24 hours after the fever began. Only 33 (12.0%) will wait longer than two days, while three caregivers (1.1%) report not taking their child to the doctor at all for fever (Figure 3).

When selecting a location that the caregiver prefers the child be treated for a fever, 45.3% (125) prefer to take their child to the pediatrician’s office. The second most common setting that was chosen as a preferred location was the ED (26.8%). Both the pediatrician’s office and the ED were the first choice of 35 caregivers (12.7%). Of note, a total of 109 (39.5%) caregivers included the ED as at least one of their preferred choices to take their child. Only 6.9% (19) of caregivers solely selected urgent care as their preferred location to take their febrile child. 

Caregivers were polled on their preferred methods for treating their child when it is determined they are febrile. Management with medicine alone was chosen by 176 (63.8%), while 67 (24.3%) would give a cool bath in addition to administering medicine. Concerning the medication administered, 123 (44.6%) caregivers reported acetaminophen as their preferred lone treatment and 49 (17.8%) chose ibuprofen. Both acetaminophen and ibuprofen were chosen by 79 (28.6%) caregivers. Only four (1.5%) reported not administering any medication to their febrile child.

The caregivers were polled regarding their greatest worries when their child becomes febrile with dehydration was the most common concern (21.0%) followed by seizures (5.8%) and brain damage (3.3%). Unspecified worries were held by 29 caregivers (10.5%). Many caregivers (108) (39.1%) were worried about any combination of the above three symptoms. Surprisingly, 29 caregivers (10.5%) had no concerns when their child has a fever.

## Discussion

Our study shows that a variety of detection and management methods for fever in children by caregivers exists in all domains studied. Although fever is typically a normal physiologic response to non-emergent illnesses, it produces anxiety in caregivers that has persisted since Schmitt first published his study on “fever phobia” in 1980 [3]. The fears surrounding pediatric fever causes caregivers to take unnecessary actions that are potentially harmful to the child.

In our study, most (91.0%) caregivers possessed a thermometer at home, and about a third will use it to check the child’s temperature simply when they feel warm, disregarding other reliable measures of systemic illness such as altered mental status [9]. We also found that about one-third of caregivers surveyed believed the fever threshold to be 99.5°F, and an additional 15.6% believed their child to have a fever “when they felt hot”. A study by Poirier et al. in 2010 [2] found the median temperature at which pediatric caregivers believed a fever to be was 100.4°F. In 2001, Crocetti et al. investigated “fever phobia” in two urban hospitals in Baltimore (Maryland, USA) and found that nearly 50% of caregivers believed a high fever to be between 100.4-102°F. 

According to our results, caregivers may not be using the recommended methods for obtaining an accurate temperature, especially in children 5 years or younger. The guidelines recommend rectal temperatures for children under 5 years old with the armpit or ear being the next appropriate site for an accurate reading [10]. Only 34.6% of caregivers in our study with children under 5 years old reported using the rectal route as one of their methods to obtain a temperature. A 2015 meta-analysis that compared peripheral temperatures with central temperatures found the peripheral temperatures to be clinically inaccurate and unacceptable to use when the temperature is influencing clinical decisions [11]. Inaccurate readings in younger children due to lack of education regarding proper techniques could be helping to induce “fever phobia” in caregivers.

Correctly identifying a fever and accompanying serious signs of illness is of the utmost importance so as to not overuse available healthcare resources. The vast majority (87.0%) of caregivers, though, report they will seek care for a febrile child between 0 and 2 days of onset. According to Berry et al., 58 to 82% of all pediatric ED visits were categorized as “non-urgent” [12]. This was further studied in an urban pediatric ED by Kubicek et al., and they found 63% of caregivers assessed their child as having an urgent condition despite being triaged by medical professionals at the lowest level of acuity [13]. There are significant financial implications for our already strained healthcare system due to this discordance. According to a statistical analysis of pediatric ED usage by the Agency for Healthcare Research and Quality, in 2015, children accounted for over 20% of all ED visits in the United States. Of the 30 million total visits by children aged 18 or younger, 97% were treated and released without admission [14]. This data suggests parents are not well-educated on identifying serious illness in their children and instead may act on fear when deciding to seek treatment. The ED is often utilized for non-life-threatening conditions such as fever, as our data showed 39.5% of caregivers included the ED as a preferred choice for their child to be evaluated. The excessive usage of emergency resources puts additional strain on our healthcare system. The average office-based visit costs $187 versus $767 for an ED-based visit according to a 2007 report from The Network for Excellence in Health Innovation (NEHI). In this report, NEHI utilized the cost difference in ED vs. office visits ($580) multiplied by the number of unnecessary ED visits and estimated that $38 billion were spent on avoidable ED visits in 2007 alone [15].

Similar worries held by caregivers regarding pediatric fever have persisted in literature for decades. According to Schmitt’s data from 1980, the majority were most concerned about brain damage due to a fever [3]. Seizures were the most commonly raised concern in Crocetti’s 2001 caregiver population with brain damage being the second most common [4]. In both studies, 4% of caregivers were most concerned about dehydration in a febrile child [3-4]. With 39% of caregivers in our study worried about some combination of dehydration, seizures, and/or brain damage, it is clear that baseless fears are driving the inappropriate management. Interestingly, over 10% of caregivers that brought their child to the ED for fever in our study reported having no accompanying concerns, suggesting a doctor visit for fever could be an impulsive decision for caregivers.

Treatment of pediatric fever is also frequently misunderstood by the pediatric caregiver population. Reducing the amplitude of a fever in a generally healthy child does not necessarily reduce morbidity or mortality risk; therefore, the actual goal of antipyretic therapy should be to improve comfort level instead of reducing body temperature [1]. Combination therapy with acetaminophen and ibuprofen is effective in reducing fever, but there are risks of dosing errors and adverse reactions. Consequently, it is recommended that caregivers aim to improve the child’s comfort level versus treating the number on the thermometer. According to the 2017 annual reports of the American Association of Poison Control Centers' National Poison Data System [16], 17% of dosing errors for antipyretics occurred in children <5 years of age and 9.6% in children between the ages of 6-12. Additionally, acetaminophen and aspirin were among the top generic products that caused fatalities. Nearly one-third of the caregivers we surveyed prefer to alternate acetaminophen and ibuprofen when treating a fever. Most caregivers (44.6%) preferred only to give acetaminophen while 17.8% preferred ibuprofen as their sole treatment of choice. When polled on their preferred methods for treating their febrile child, 176 (63.8%) caregivers would give medicine only, while 67 (24.3%) would give a cool bath in addition to administering medicine. Despite the risk of acetaminophen and ibuprofen toxicity, and the recommendations to solely treat according to comfort level, many pediatric caregivers are quick to treat fever.

This study had a variety of limitations. It was performed only on those caregivers presenting to the ED, which may have heightened their anxiety and influenced answers. It was also limited in that it lacked inquiries into access to care, home resources, specific education levels, or caregivers and demographic information. This could be evaluated in future studies. It was also a preliminary study meant to provide data for a future educational intervention in which we planned to provide intervention in the form of an educational pamphlet to pediatric caregivers who present to the ED with a plan for follow-up phone calls at six months and 12 months after intervention to re-evaluate their comfort with handling pediatric fevers utilizing the same survey. 

## Conclusions

Our study found caregiver knowledge gaps in the identification of fever as well as specific concerns that fever would lead to dehydration and severe infection. These concerns led to seeking care very early in a child's illness in both the emergency department and pediatrician's office despite literature supporting little to no risk or morbidity or morality in most children with febrile illnesses. This presents an opportunity for further caregiver education to decrease or alter the timing or location of care sought in a pediatric febrile illness.

## References

[1] McDermott KW, Stocks C, Freeman WJ (2006 Feb-). Overview of pediatric emergency department visits, 2015: statistical brief #242. Healthcare Cost and Utilization Project (HCUP) Statistical Briefs [Internet].

[2] Sullivan JE, Farrar HC (2011). Fever and antipyretic use in children. Pediatrics.

[3] Poirier MP, Collins EP, McGuire E (2010). Fever phobia: a survey of caregivers of children seen in a pediatric emergency department. Clin Pediatr (Phila).

[4] Schmitt BD (1980). Fever phobia: misconceptions of parents about fevers. Am J Dis Child.

[5] Crocetti M, Moghbeli N, Serwint J (2001). Fever phobia revisited: have parental misconceptions about fever changed in 20 years?. Pediatrics.

[6] Rupe A, Ahlers-Schmidt CR, Wittler R (2010). A comparison of perceptions of fever and fever phobia by ethnicity. Clin Pediatr (Phila).

[7] Clericetti CM, Milani GP, Bianchetti MG (2019). Systematic review finds that fever phobia is a worldwide issue among caregivers and healthcare providers. Acta Paediatr.

[8] Lubrano A (2020). New census figures on Philly neighborhoods show inequality, high numbers of whites living in poverty. https://www.inquirer.com/philly/news/poverty-new-census-figures-philadelphia-neighborhoods-whites-opioids-20181206.html-2.

[9] Zampieri FG, Park M, Machado FS, Azevedo LC (2011). Sepsis-associated encephalopathy: not just delirium. Clinics (Sao Paulo).

[10] (2000). How to take a child's temperature. Paediatr Child Health.

[11] Niven DJ, Gaudet JE, Laupland KB, Mrklas KJ, Roberts DJ, Stelfox HT (2015). Accuracy of peripheral thermometers for estimating temperature: a systematic review and meta-analysis. Ann Intern Med.

[12] Berry A, Brousseau D, Brotanek JM, Tomany-Korman S, Flores G (2008). Why do parents bring children to the emergency department for nonurgent conditions? a qualitative study. Ambul Pediatr.

[13] Kubicek K, Liu D, Beaudin C, Supan J, Weiss G, Lu Y, Kipke MD (2012). A profile of nonurgent emergency department use in an urban pediatric hospital. Pediatr Emerg Care.

[14] Moore BJ, Stocks C, Owens PL (2020). Trends in emergency department visits, 2006-2014. HCUP statistical brief #227. Healthcare Cost and Utilization Project (HCUP) Statistical Briefs [Internet].

[15] (2020). A matter of urgency: reducing emergency department overuse. A NEHI research brief - March 2010. https://www.nehi.net/writable/publication_files/file/nehi_ed_overuse_issue_brief_032610finaledits.pdf.

[16] Gummin DD, Mowry JB, Spyker DA, Brooks DE, Osterthaler KM, Banner W (2018). 2017 annual report of the American Association of Poison Control Centers' National Poison Data System (NPDS): 35th annual report. Clin Toxicol (Phila).

